# Evaluation of the antibacterial activity of cinnamon essential oil and its individual compounds on *Aggregatibacter actinomycetemcomitans* isolated from black extrinsic tooth stain: an in vitro study

**DOI:** 10.1007/s40368-023-00841-y

**Published:** 2023-09-25

**Authors:** W. A. Lotfy, M. A. Matar, B. M. Alkersh

**Affiliations:** 1 Department of Microbiology, Faculty of Dentistry, Pharos University in Alexandria, Alexandria, Egypt; 2 Department of Pediatric Dentistry, Faculty of Dentistry, Pharos University in Alexandria, Alexandria, Egypt; 3 Marine Environment Division, Marine Microbiology Laboratory, National Institute of Oceanography and Fisheries, Alexandria, Egypt

**Keywords:** *Aggregatibacter actinomycetemcomitans*, Black extrinsic tooth stain, Cinnamon oil, Antibacterial

## Abstract

**Aim:**

Black extrinsic tooth stain (BETS) is a health challenge that commonly affects children. *Aggregatibacter actinomycetemcomitans* (*Aa*) presents in higher prevalence within the polymicrobial community of BETS. In this study, the anti-planktonic and anti-sessile activities of cinnamon essential oil (CEO) and its individual compounds against *Aa* were evaluated. The preventive effect of CEO and its active substances on BETS formation was also studied in vitro.

**Methods:**

*Aa* was isolated from a preschool child with BETS and was identified based on the morphological characteristics, MALDI-TOF mass spectroscopy and 16S rRNA sequencing. The effect of CEO and its individual compounds on the growth kinetics of planktonic and sessile *Aa* cells as well as their antibacterial efficacy and their rate of bacterial killing were examined. The preventive effect of CEO and its active substances on the formation of BETS was evaluated using an ex vivo model. The data were analysed using one-way analysis of variance (ANOVA) and the significance level was set at *p* < 0.05.

**Results:**

Out of eight individual compounds of CEO, only eugenol, cinnamaldehyde and α-methyl cinnamaldehyde showed anti-*Aa* activities. The values of the minimum inhibitory concentrations (MICs) were in the following order: CEO (421.5 mg/ml) > α-methyl cinnamaldehyde (26.37 mg/ml) > cinnamaldehyde (0.209 mg/ml) > eugenol (0.052 mg/ml). CEO, eugenol, cinnamaldehyde and α-methyl cinnamaldehyde, respectively, exhibited two-, four-, four- and eightfold increase of sessile MIC compared to their planktonic MIC. The growth kinetics of both planktonic and sessile *Aa* in the presence of CEO, eugenol, cinnamaldehyde and α-methyl cinnamaldehyde revealed a complete inhibition at the MICs and 5.3%–37.4% biofilm inhibition at sub-MICs. The time-killing study demonstrated that CEO, eugenol and cinnamaldehyde were capable of reducing the survival rate of both planktonic and sessile *Aa* cells after 15–20 and 25–30 min, respectively. However, α-methyl cinnamaldehyde showed a superior anti-planktonic to anti-biofilm activity. The daily incorporation of CEO, eugenol and cinnamaldehyde at their MICs for 14 days totally prevented the formation of BETS in the ex vivo model; however, in the case of α-methyl cinnamaldehyde, BETS was visually detectable after 10 days.

**Conclusion:**

CEO and its individual compounds have marked antibacterial activity against *Aa*. The effective results against planktonic and sessile *Aa* within reasonable time indicate that they can be used to prevent BETS.

## Introduction

Black extrinsic tooth stain (BETS) is widespread amongst children and affects the aesthetics of teeth (Li et al. 2015). It is distinguished by dark dots or dark lines on the cervical third of enamel in both deciduous and permanent teeth (Koch et al. 2001). BETS is difficult to be wiped off by daily tooth brushing, it can be removed through ultrasonic scaling, polishing and fluoride pumice (Ronay and Attin 2011). Moreover, the cleaning procedure required for its removal can be challenging to dentists, especially pit and fissure BETS (Ronay and Attin 2011). Furthermore, it tends to recur even with good oral hygiene after scaling (Hattab et al. 1999). BETS comprises insoluble ferric sulphide which is formed by the reaction between hydrogen sulphide that is produced by bacteria and the salivary iron (Li et al. 2015).

Previous studies proposed chromogenic bacteria as the causative agents of BETS formation. *Porphyromonas gingivalis*, *Prevotella intermedia*, *Prevotella melaninogenica* and *Prevotella nigrescens* are also closely related to BETS (França-Pinto et al. 2012; Slots 1974; Soukos et al. 2005). *Aggregatibacter actinomycetemcomitans* (*Aa*) is a Gram-negative, facultative anaerobic coccobacillus bacterium of the Pasteurellaceae family (Rahamat‐Langendoen et al. 2011). *Aa* is implicated in aggressive periodontitis in a subset of African and Middle Eastern patients (Fine et al. 2018). Saba et al. (2006) reported the presence of significantly higher prevalence of *Actinomyces* and *Aa* in BETS. Sakai et al. (2007) also detected a high prevalence of *Aa* in the saliva of children with mixed dentition. However, the association of *Aa* in the formation of BETS has not yet been documented. Here, we aim at revealing whether *Aa* is related to BETS formation or not.

There is a lack of reports in the dental literature that focus on the treatment and prevention of BETS. Particularly, the unique microbiota of BETS necessitates an antibacterial agent that is capable of inhibiting the growth of the etiological agent. On the other hand, seeking alternative agents allowing for the use of plant-derived essential oils to promote dental health is of particular interest (Pourabbas and Delazar 2010). Hence, the development of a natural antibacterial agent that is safe for the host as well as specific for BETS microbiota is awaited. In a recent study, Arpag et al. (2020) stated that the antibacterial effect of *Hypericum perforatum* essential oil on *Porphyromonas gingivalis* and *Aa* strains increased synergistically when combined with chlorhexidine. Despite considering chlorhexidine as an effective chemotherapy in controlling plaque flora (Ronanki et al. 2016), taste altering and tooth staining limit its prolonged administration. Lavine et al. (2018) also reported that virgin coconut oil reduced the viability of *Actinomyces* sp. isolated from children aged 4–11 years with BETS.

*Cinnamomum zeylanicum* belongs to the family Lauraceae; cinnamon essential oil (CEO) is rich in transcinnamaldehyde which has antimicrobial effects against various pathogens. More than 300 volatiles have been found to be the constituents of CEO (Malsawmtluangi et al. 2016). El Atki et al. (2019) demonstrated a synergistic interaction of CEO and chloramphenicol or ampicillin against *Staphylococcus aureus* and against *Escherichia coli*. Further, the combination of CEO and streptomycin showed additive effects against *Staphylococcus aureus*, *Escherichia coli* and *Pseudomonas aeruginosa*. However, their study did not evaluate the effect of CEO on oral bacteria. In another study, Panjaitan et al. (2022) reported that the ethanolic extract of cinnamon with a concentration of 7.5% and 2.5%, respectively, showed effective influence against biofilm formation of *Porphyromonas gingivalis* and *Aa*. Nevertheless, their study focused on the antimicrobial effect of the ethanolic extract of cinnamon which differs from CEO with respect to the chemical composition, physical properties, solubility and antibacterial activity.

The aims of this work are: (i) to investigate the association of *Aa* in BETS formation, (ii) to assess the antibacterial efficacy of CEO and its active compounds against planktonic and sessile *Aa* that has been isolated from BETS and (iii) to evaluate the preventive effect of CEO and its active substances on the formation of BETS using an ex vivo model. To our knowledge, no single report has addressed the in vitro effect of CEO on the formation of BETS and on *Aa* isolated from BETS.

## Materials and methods

### Media and chemicals

The following media were used throughout this study: viability-maintaining microbiostatic medium, anaerobically prepared (VMGA III) transport medium (Doan et al. 1999), tryptone soya serum bacitracin vancomycin agar (TSBV, HiMedia Laboratories, India) (Slots 1982), tryptone soya broth (TSB, Biolife, Italy), tryptone soya agar (TSA, Biolife, Italy), Müller–Hinton broth (MHB, Biolife, Italy) and Müller–Hinton agar (MHA, Biolife, Italy). All chemicals used in this work were of analytical grade (HiMedia Laboratories, India). CEO was purchased from Frey & Lau GmbH, Henstedt-Ulzburg, Germany.

### Chemical analysis of CEO

The chemical analysis of CEO was carried out according to the method described by Witasari et al. (2022). A volume of 1 μl of CEO was injected into gas chromatograph–mass spectrometer (GC–MS, Thermo Agilent Technologies-7890A) with Helium as the carrier gas in Trace Gold System Qualification Column (TG-SQC). Analysis of samples was carried out as follows: initial temperature 50 °C for 1 min, 250 °C for 5 min and finally 290 °C for 2 min. Mass spectral range was set at 40–1000 Hz and the samples were injected in split mode constant flow 1.5 ml/min. Ion source temperature and mass transfer line temperature were 300 °C. The mass spectra of volatiles were compared to those in the library of MS database and with those of standard substances for identification of individual volatiles. The amount of each individual compound of CEO was calculated and expressed as a percentage of the peak area relative to the total peak area obtained.

### Sample collection and isolation of *Aa*

All methods were carried out in accordance with The Code of Ethics of Pharos University in Alexandria for experiments involving human subjects. All experimental protocols were approved by the Ethical Committee of Pharos University in Alexandria (PUA0220233263068). Samples were collected at Pharos University dental clinic from a 4-year-old male child diagnosed with BETS after obtaining an informed consent from his parents. The selected subject had no antibiotic therapy during the previous 1 month, had no orthodontic treatment and had no intraoral prosthesis or intraoral pathology. The selected child was asked to rinse his mouth with water before sample collection. Using a sterile curette, samples of BETS were scraped from the black area of the vestibular and lingual surfaces of the teeth and transferred to 10 ml autoclaved screw capped test tubes containing 5 ml of VMGA III transport medium (Doan et al. 1999). The tubes were immediately transported to the laboratory in an ice box for cultivation. Each tube was mixed thoroughly by vortex mixer; thenceforward, an aliquot of 1 ml was plated onto sterile TSBV agar medium for a selective isolation of *Aa* (Slots 1982). After anaerobic incubation at 37 °C for 72 h, the obtained bacterial colonies were purified by streaking on TSBV agar medium.

### Identification of the obtained bacterial isolate

The obtained isolate was first stained by Gram stain and examined under light microscope 100 × (Leica DM300, Danaher, USA). This was followed by matrix-assisted laser desorption/ionisation time-of-flight mass spectrometry (MALDI-TOF MS) identification and 16S rRNA sequencing. The proteomic-based spectrum generated by MALDI-TOF MS (Reflex Bruker Daltonics, Germany) was compared against the reference spectra using Bruker MALDI-TOF Biotyper software to achieve a confidence score for the most related species (Randall et al. 2015). On the other hand, DNA was extracted from the isolate, 16S rRNA gene was amplified and purified to remove primers, nucleotides, enzymes, and other impurities using QIAquick PCR purification kit (Qiagen, USA) according to the method explained by (Riggio et al. 1996). DNA sequences were obtained using an ABI PRISM 377 DNA sequencer and ABI PRISM Big dye Terminator Cycle Sequencing (Perkin Elmer). The nucleotide sequence of the isolate was compared with similar sequences on the NCBI BLAST database www.ncbi.nlm.nih.gov/blast. The multiple sequence alignment and molecular evolutionary analysis were performed using MEGA version 6 software (Tamura et al. 2013).

### Determination of the antibacterial activity of CEO against *Aa*

A seed culture of *Aa* (10^5^ cfu/ml) was inoculated on the surface of MHA medium using a right-angled glass spreader and the agar well diffusion method (Atlas 1997) was adopted. An aliquot of 10 μl of CEO or individual compounds was placed at the centre of each well (6 mm diameter and 4 mm depth). Ceftriaxone at a final concentration of 0.001 mg/ml was used as a positive control. The agar plates were incubated in an anaerobic jar for 24 h at 37 °C. After incubation, the diameter of inhibition zone was measured by Vernier calliper (Mitutoyo 500-195).

### Biofilm production assay

The ability of *Aa* to form biofilm was evaluated as described elsewhere (Erriu et al. 2012). Briefly, biofilms were formed by adding 100 μl of *Aa* suspension in 100 μl TSB medium in each microtiter plate wells. TSB was removed after anaerobic incubation at 37 °C for 48 h. The wells were washed three times with sterile phosphate buffer saline (PBS) to remove non-adherent cells. The wells were allowed to dry for 15 min prior staining with 200 μl of 0.4% crystal violet for 15 min. The unbound stain was removed and the wells were washed three times with PBS then air dried for 15 min. An aliquot of 200 μl of 33% acetic acid was used to solubilise the remaining crystal violet in each well. Absorbance was measured at 620 nm using a microplate reader (MR-96, Clindiag Systems Co. LTD., China).

### Antimicrobial activity assay

The antimicrobial activity of CEO and its individual bioactive compounds was evaluated as described by Vanegas et al. (2021) with a few modifications. Briefly, various concentrations of CEO or its individual bioactive compounds were prepared by mixing the agent with tween 80 and double-distilled water. The reaction mixture was prepared in a 96-well microtiter plate; each well contains 50 μl inoculum (10^5^ cfu/ml), 50 μl of different CEO concentrations and 50 μl of MHB medium. Ceftriaxone at a final concentration of 0.001 mg/ml was used as a positive control (Bhat et al. 2019) and test agent-free wells served as negative control. The contents of each well were mixed thoroughly and then incubated anaerobically at 37 °C for 24 h. To evaluate the minimum inhibitory concentration (MIC) value, the growth of *Aa* was determined by measuring the optical density (OD) at 600 nm using a microplate reader (MR-96, Clindiag Systems Co. LTD., China) and confirmed by plating 5 μl samples from clear wells on MHA medium. The MIC was defined as the dose that produced a 90% reduction of *Aa* growth compared with the growth of the agent-free control (Frassinetti et al. 2011). To determine the MIC of CEO and its fractions in sessile conditions, the previous steps were performed on *Aa* biofilm as described under biofilm production assay.

### Inhibition of *Aa* growth and biofilm formation by CEO and its individual components

The inhibition of *Aa* growth and biofilm formation by CEO and its bioactive compounds was determined as described elsewhere (Martínez et al. 2021). *Aa* suspension was adjusted to 10^5^ cfu/ml and the experiment was set as mentioned in the preceding section. Planktonic *Aa* was cultured on TSB medium, incubated under anaerobic conditions for 48 h at 37 °C in the presence or absence of test agents. The concentrations of test agents were based on the MIC values against planktonic *Aa* cells. Ceftriaxone at a final concentration of 0.001 mg/ml was used as a positive control and test agent-free wells served as negative control. The OD at 600 nm was monitored at each time interval of sampling. The survival of cells was verified by a plate counting on TSA medium after 48 h of anaerobic incubation at 37 °C. Biofilms were allowed to form as described previously and various concentrations of test agents were added based on the MIC values against sessile *Aa* cells for 24 h. Subsequently, the biofilm formation was evaluated as described previously and the inhibition of biofilm formation was calculated according to the following equation: The biofilm inhibition (%) = (Mean OD_620_ of test /Mean OD_620_ of control) × 100.

### Time-kill determination of planktonic and sessile *Aa*

The time-kill assay of planktonic and sessile *Aa* was performed as explained by Vasconcelos et al. (2023) with a few modifications. Briefly, an aliquot of 30 ml TSB medium containing 10^2^ or 10^5^ cfu/ml *Aa* was grown in 100 ml flasks at 37 °C under anaerobic conditions with or without the test agents at their MIC. The OD at 600 nm was measured at 0, 5, 10, 15, 20, 25 and 30 min using spectrophotometer (PD-303, APEL Co., Japan). The colony counts were also determined based on a standard curve of OD as a function of *Aa* concentration (cfu/ml). To determine the time-kill of sessile *Aa* cells, the biofilms of *Aa* were allowed to form as described previously. Test agents were added at their MIC to the formed biofilm; then, sessile *Aa* cells were scraped with PBS and vigorously vortexed to be disaggregated at 0, 5, 10, 15, 20, 25 and 30 min. Total viable count of each well was quantified on TSA after incubation for 24 h at 37 °C under anaerobic conditions. Test agent-free wells were treated as negative control and ceftriaxone at a concentration of 0.001 mg/ml was used as a positive control.

### Evaluation of BETS inhibition by CEO and its individual components in an ex vivo model

The inhibition of BETS formation by CEO and its bioactive compounds was evaluated in an ex vivo model using healthy fallen deciduous teeth sterilised by autoclaving. The experimental protocol was performed as described by Conti et al. (2002) with few modifications using the isolated *Aa* strain, *Prevotella intermedia* ATCC 15032, and *Porphyromonas gingivalis* ATCC 33277. Briefly, three teeth were used for each experimental group, each tooth was submerged in 3 ml sterile human saliva with TSB medium and inoculated with 10^5^ cfu/ml *Aa*. The preparation was incubated at 37 °C under anaerobic conditions with or without (negative control) the test agents at their sessile MIC. Starting from the third day after inoculation of *Aa*, the test agents were added to the preparations at their MIC with a medium change every 24 h for 14 days. After the incubation period and under aseptic conditions, each tooth was washed with 10 ml sterile saline and then scraped with a sterile curette for 6 min using 6 ml of sterile saline to remove sessile cells and BETS. An aliquot of 100 μl of appropriate dilutions of the suspensions obtained after scraping was inoculated onto the surface of TSA medium. The survival of cells was verified by counting the number of colonies in each plate after 48 h of anaerobic incubation at 37 °C.

### Statistical analysis

Each experiment was carried out three times in triplicate; the mean and standard deviation were calculated using Microsoft Excel 2010. Data were analysed using IBM SPSS software package version 20.0*.* (Armonk, NY: IBM Corp). The Kolmogorov–Smirnov test was used to verify the normality of distribution and one-way analysis of variance (ANOVA) was used to determine the significance level at *p* < 0.05.

## Results

### Chemical analysis of CEO using GC–MS

**Fig. 1:**
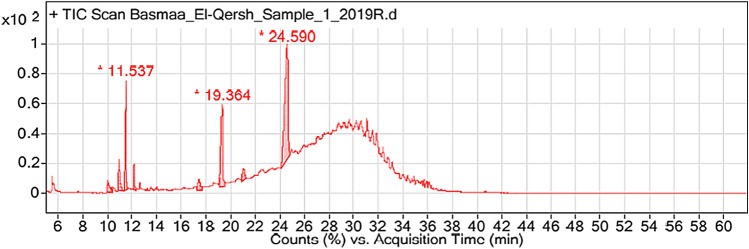
GC–MS chromatogram showing the ingredients of CEO

The chemical analysis of CEO using GC–MS has identified eight compounds (Fig. 1) constituting 100% of the total volatile oil as shown in Table 1.

**Table 1:** Chemical composition of CEO identified by GC–MS

Peak	Retention time	Retention index	Compound	Value in %
1	10.036	2009	Cinnamaldehyde	3.32
2	10.959	1209	α-Methyl cinnamaldehyde	5.23
3	11.537	2197	Eugenol	13.78
4	12.182	1225	Benzoic acid 4-isopropyl ethyl ester	2.55
5	17.402	1687	Methyl isomyristate	3.74
6	19.364	1988	n-Butyl myristate	20.79
7	21.079	2152	17-Octadecynoic acid methyl ester	3.32
8	24.59	2140	9,12-Octadecadienoyl chloride	47.27

### Characterisation and identification of *Aa* isolated from BETS

**Fig. 2:**
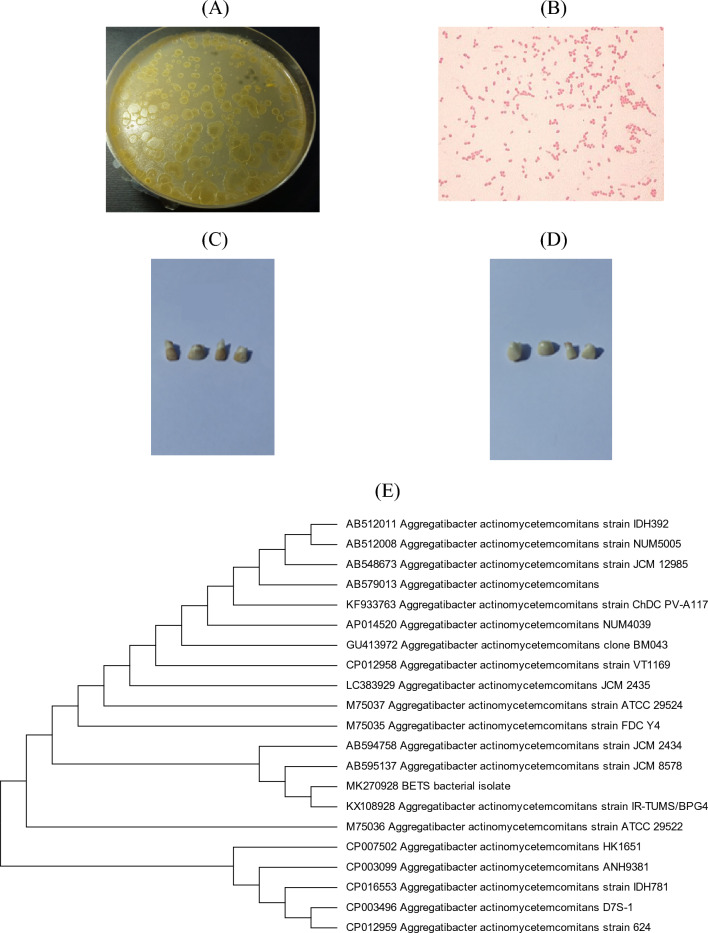
Cultural (**A**) and microscopic (**B**) examination of the BETS bacterial isolate. Induction of BETS in sound deciduous teeth by *Aa* isolate (**C**) versus control teeth (**D**). Phylogenetic analysis of the BETS bacterial isolate based on the sequence of 16S rRNA by maximum likelihood method (**E**)

The colonies of the isolate on TSBV appeared as convex, circular, glistening, translucent, with star-like inner structure and slightly irregular edges (Fig. 2A). Accordingly, the isolate was primarily identified as *Aa* (Slots 1982). The microscopic examination of these colonies has revealed a Gram-negative coccobacillus bacterium (Fig. 2B). The isolate was capable of inducing BETS in sound fallen deciduous teeth after sterilisation by autoclaving then incubation in TSB medium for 6 weeks at 37 °C (Fig. 2C); whereas, no BETS formation was observed in control teeth (Fig. 2D).

MALDI-TOF MS-based proteomic identification was used to compare the cellular protein profiles of the bacterial isolate to reference spectra. According to the results obtained from this analysis (Table 2), the bacterium isolated from BETS recorded high score values (2.5–2.68) to *Aa* which indicated a highly probable identification.

**Table 2:** MALDI-TOF MS analysis of the BETS bacterial isolate

Best match	Score value*	Second best match	Score value*
*Aggregatibacter actinomycetemcomitans*	2.53	*Aggregatibacter actinomycetemcomitans*	2.11
*Aggregatibacter actinomycetemcomitans*	2.68	*Aggregatibacter actinomycetemcomitans*	2.10
*Aggregatibacter actinomycetemcomitans*	2.50	*Aggregatibacter actinomycetemcomitans*	2.08

*2.300–3.000, highly probable species identification; 2.000–2.299, probable species identification; 1.700–1.999, probable genus identification and 0.000–1.699, not reliable identification

The rRNA fragment of the experimental bacterium showed 100% identity to the homologous fragments of *Aa*. The sequence was deposited in the GenBank under accession number MK270928. The phylogenetic analysis of the 16S rRNA sequences was constructed by applying the maximum likelihood method as shown in Fig. 2E. The experimental bacterial isolate was closely related to *Aa* and was deposited in the Egyptian Microbial Culture Collection Network, Alexandria, Egypt (accession number EMCCN 4253).

### The antibacterial activities of CEO and its individual components against *Aa* under planktonic and sessile conditions

**Fig. 3:**
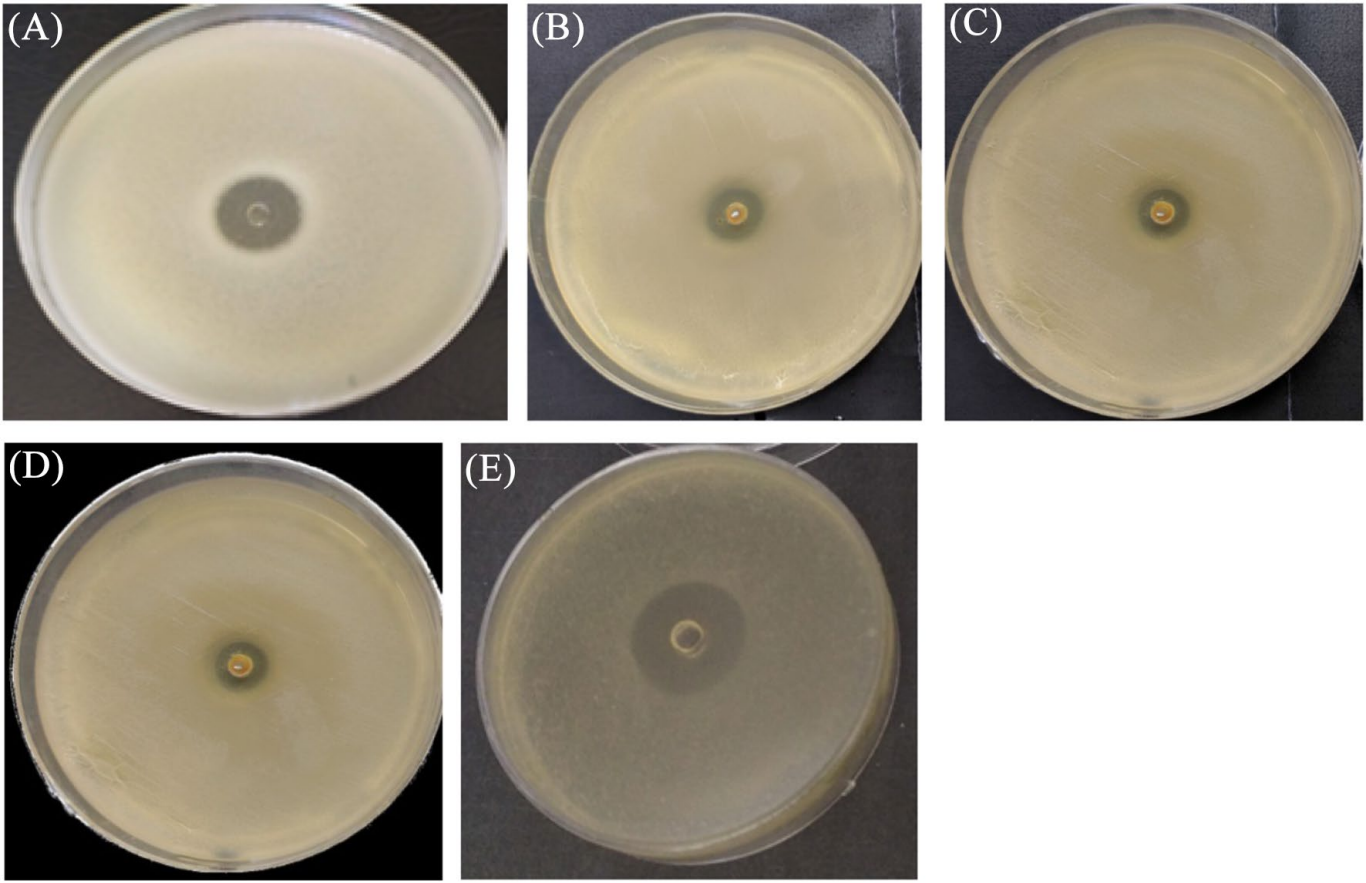
Inhibition zone of *Aa* around the well that was initially filled with CEO (**A**), eugenol (**B**), cinnamaldehyde (**C**), α-methyl cinnamaldehyde (**D**) and ceftriaxone (**E**)

*Aa* was used to evaluate the anti-planktonic and anti-sessile activities of CEO and its individual bioactive compounds. Out of eight individual fractions of CEO, only eugenol, cinnamaldehyde and α-methyl cinnamaldehyde demonstrated anti-*Aa* activities (Fig. 3). The inhibition zones and MICs of CEO and its individual components against *Aa* are shown in Table 3. The results on the inhibitory effect of CEO, eugenol, cinnamaldehyde, α-methyl cinnamaldehyde and negative control against *Aa* were 24.17, 18.76, 16.48, 12.13 and 0.0 mm, respectively. Moreover, the inhibition zones of CEO, eugenol, cinnamaldehyde and α-methyl cinnamaldehyde, respectively, represented 95.57%, 74.18%, 65.16% and 47.96% of the inhibition zones observed by ceftriaxone. As evident from Table 3, eugenol exhibited the lowest MIC (0.052 mg/ml) and CEO exhibited twofold increase of sessile MIC compared to its planktonic MIC. A fourfold increase of sessile MIC was also observed with eugenol and cinnamaldehyde. On the other hand, ceftriaxone and α-methyl cinnamaldehyde showed several-fold increase in sessile MIC compared to their planktonic MIC.

**Table 3:** MICs of CEO and its individual fractions against *Aa* compared to ceftriaxone under planktonic and sessile conditions

Compound	Inhibition zone of *Aa* (mm)	Planktonic MIC (mg/ml)	Sessile MIC (mg/ml)
Cinnamon oil	24.17 ± 0.76	421.5	843
Eugenol	18.76 ± 0.42	0.052	0.2
Cinnamaldehyde	16.48 ± 0.83	0.209	0.836
α-Methyl cinnamaldehyde	12.13 ± 0.26	26.37	210.96
The rest of individual fractions	0.0	–	–
Ceftriaxone	25.29 ± 0.47	0.001	0.016
Negative control	0.0	–	–

### Effects of CEO and its individual fractions on growth kinetics of *Aa* under planktonic and sessile conditions

**Fig. 4:**
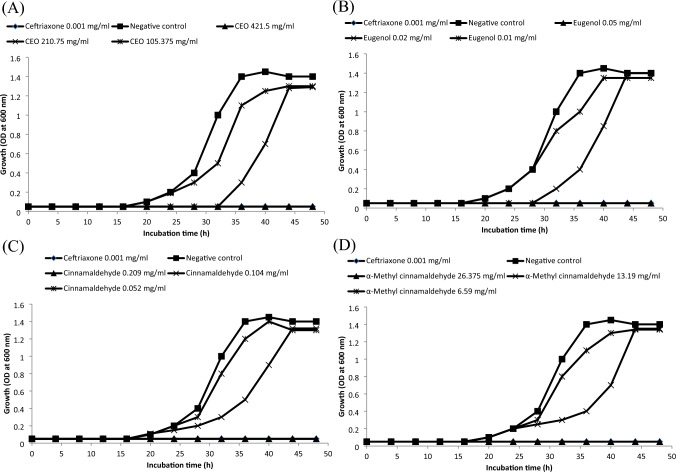
Growth kinetics of *Aa* in the absence or presence of increasing concentration of CEO (**A**), eugenol (**B**), cinnamaldehyde (**C**) and α-methyl cinnamaldehyde (**D**)

The growth kinetics of *Aa* in the presence or absence of test agents is shown in Fig. 4. The growth of *Aa* in the absence of test agents (negative control) was detected at 20 h of incubation, then reached a maximum at 40 h and was completely inhibited by the ceftriaxone concentration of 0.001 mg/ml (positive control). A remarkable antibacterial activity was observed at the CEO level of 210.75 mg/ml. At this concentration, the growth of *Aa* was postponed for 36 h. *Aa* was completely inactivated at the CEO concentration of 421.5 mg/ml. For eugenol, a noticeable inhibitory effect was detected in the presence of 0.02 mg/ml with a delay in *Aa* growth up to 32 h. Moreover, a concentration of 0.05 mg/ml was required for complete inactivation of *Aa*. On the other hand, no delayed growth of *Aa* was observed with various concentrations of cinnamaldehyde or α-methyl cinnamaldehyde. Marked anti-*Aa* effects were detected at cinnamaldehyde and α-methyl cinnamaldehyde levels of 0.104 and 13.19 mg/ml, respectively. *Aa* could be completely inhibited at cinnamaldehyde and α-methyl cinnamaldehyde concentrations of 0.209 and 26.375 mg/ml, respectively.

**Fig. 5:**
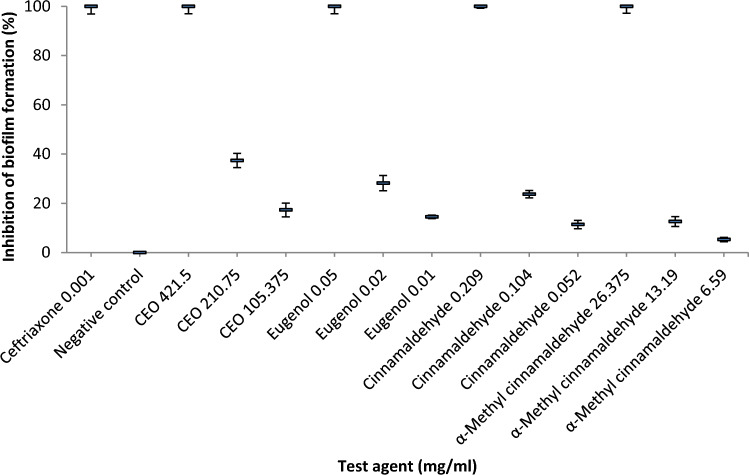
Effects of CEO, eugenol, cinnamaldehyde and α-methyl cinnamaldehyde on biofilm formation of *Aa*

On the other hand, the anti-biofilm activities of test agents at their MICs and sub-MICs are shown in Fig. 5. The ability of *Aa* to form biofilm was completely inhibited at the MICs of test agents and ceftriaxone. In the presence of half the MICs of CEO, eugenol, cinnamaldehyde and α-methyl cinnamaldehyde, biofilm formation was reduced by 37.4%, 28.2%, 23.7% and 12.6%, respectively. At one-fourth of the MICs of CEO, eugenol, cinnamaldehyde and α-methyl cinnamaldehyde, the ability of *Aa* to establish biofilm was decreased by 17.3%, 14.5%, 11.4% and 5.3%, respectively. The negative control did not show any reduction of *Aa* biofilm formation.

### Time-kill determination

**Fig. 6:**
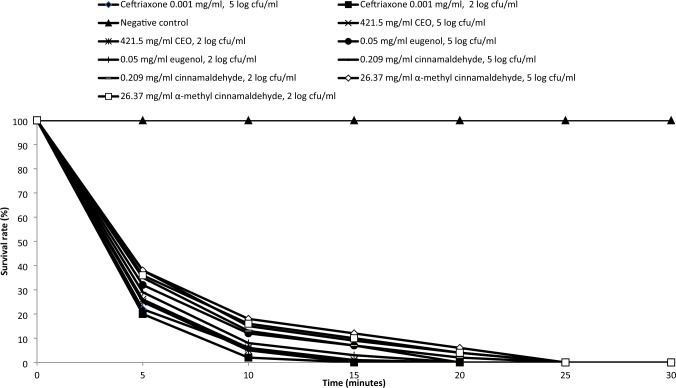
Survival rates of *Aa* in the presence of CEO, eugenol, cinnamaldehyde and α-methyl cinnamaldehyde at their MIC

To evaluate the effect of test agents on planktonic *Aa* with a rational time of regular mouth washing or food intake, the time-killing was determined. Since most infections of *Aa* occur at a low density of cells (≅ 10^2^ cfu/ml) (Fine et al. 2010), two *Aa* loads, namely 10^2^ and 10^5^ cfu/ml were tested in the current experiment. As shown in Fig. 6, within 15 min, the MIC of CEO was capable of eradicating 99% and 100% of log 5 and log 2 *Aa*, respectively. Similarly within 15 min, the MIC of eugenol could inactivate 93% and 97% of the *Aa* population with 10^5^ and 10^2^ cfu/ml, respectively. After 20 min, the MICs of cinnamaldehyde and α-methyl cinnamaldehyde eliminated 96% and 94% of log 5 *Aa*, and 98% and 96% of log 2 *Aa*, respectively. The MIC of ceftriaxone completely inhibited log 5 and log 2 *Aa* after 15 min. No significant difference was observed between CEO and ceftriaxone or eugenol and ceftriaxone (*p* < 0.05). On the other hand, the survival rate of planktonic *Aa* in the absence of test agents (negative control) was 100%.

**Fig. 7:**
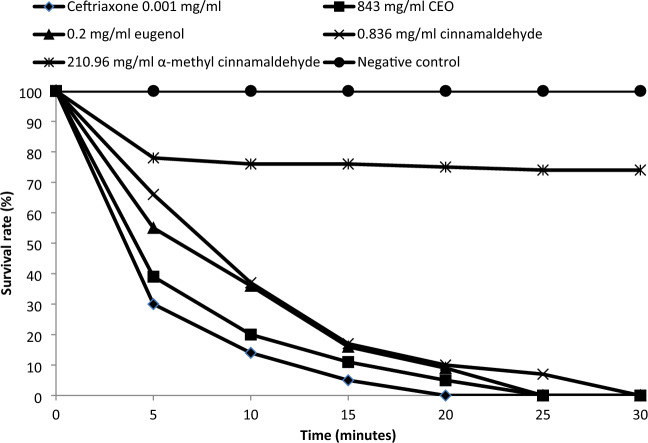
Viability of *Aa* biofilm at the sessile MIC of CEO, eugenol, cinnamaldehyde and α-methyl cinnamaldehyde

Figure 7 shows the survival rate of sessile *Aa* examined with or without the treatment of test agents and ceftriaxone at their MICs at different exposure time. After 15 min, the survival rate of sessile *Aa* was reduced to 5%, 11%, 16%, 17% and 76% by CEO, eugenol, cinnamaldehyde, α-methyl cinnamaldehyde and ceftriaxone, respectively. Ceftriaxone, CEO, eugenol and cinnamaldehyde totally inactivated the biofilm formation of *Aa* after 20, 25, 25 and 30 min, respectively. No significant difference was observed between CEO and ceftriaxone or eugenol and ceftriaxone (*p* < 0.05). On the other hand, α-methyl cinnamaldehyde was only able to reduce sessile *Aa* cells by 26% after 30 min and the survival rate of sessile *Aa* in the absence of test agents (negative control) was 100%.

### Evaluation of BETS inhibition by CEO and its individual components in an ex vivo model

**Fig. 8:**
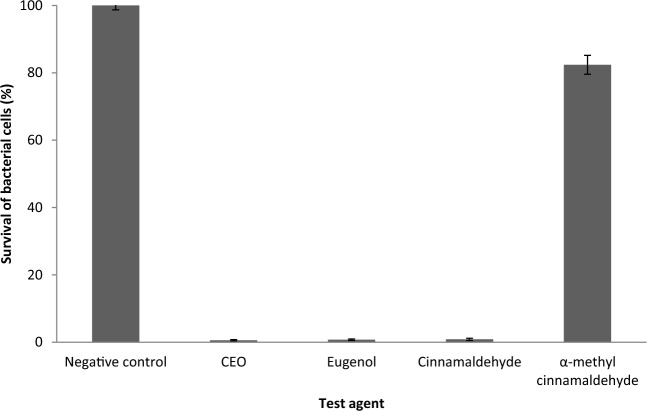
Inhibition of BETS formation on deciduous teeth in the ex vivo model by CEO, eugenol, cinnamaldehyde and α-methyl cinnamaldehyde

The effect of CEO and its individual compounds on BETS formation by the isolated *Aa* strain, *Prevotella intermedia* and *Porphyromonas gingivalis* was evaluated using an ex vivio model. BETS formation was observed on the teeth of the negative control group and the α-methyl cinnamaldehyde group after 8 and 10 days of incubation, respectively. On the other hand, BETS formation was not visually detected in the CEO, eugenol and cinnamaldehyde groups for up to 14 days. These results were confirmed by the total viable count of the mixed bacterial culture. After 14 days, the survival of the sessile cells in the mixed culture was reduced to 0.57%, 0.75%, 0.87% and 82.37% by CEO, eugenol, cinnamaldehyde and α-methyl cinnamaldehyde, respectively (Fig. 8).

## Discussion

Black extrinsic tooth stain affects the aesthetics of children’s teeth and tends to recur even after removal by conventional procedures (Hattab et al. 1999; Li et al. 2015). In the current study, *Aggregatibacter actinomycetemcomitans* was found to be associated with the in vitro formation of black extrinsic tooth stain and three individual compounds of cinnamon essential oil, namely eugenol, cinnamaldehyde and α-methyl cinnamaldehyde showed anti-*Aggregatibacter actinomycetemcomitans* activities. The sessile minimum inhibitory concentration value of cinnamon essential oil, eugenol, cinnamaldehyde and α-methyl cinnamaldehyde was also determined and, respectively, showed two-, four-, four- and eightfold increase compared to their planktonic minimum inhibitory concentration. Moreover, at the minimum inhibitory concentrations of cinnamon essential oil, eugenol, cinnamaldehyde and α-methyl cinnamaldehyde, the growth kinetics of both planktonic and sessile *Aggregatibacter actinomycetemcomitans* showed a complete inhibition. The time-killing study indicated that the survival rate of planktonic and sessile *Aggregatibacter actinomycetemcomitans* cells was reduced by cinnamon essential oil, eugenol and cinnamaldehyde after 15–20 and 25–30 min, respectively. Cinnamon essential oil, eugenol and cinnamaldehyde were also capable of preventing the formation of black extrinsic tooth stain in an ex vivo model.

*Aggregatibacter actinomycetemcomitans* is associated with aggressive periodontitis in a subset of patients from Africa and Middle East (Fine et al. 2018). In few studies *Aggregatibacter actinomycetemcomitans* has been also detected in black extrinsic tooth stain in a high prevalence (Saba et al. 2006; Sakai et al. 2007). However, the interrelation between *Aggregatibacter actinomycetemcomitans* and the formation of black extrinsic tooth stain has not been yet documented. In the current study, *Aggregatibacter actinomycetemcomitans* strain was isolated from black extrinsic tooth stain and the bacterium was identified based on the morphological characteristics, matrix-assisted laser desorption/ionisation time-of-flight mass spectrometry and 16S rRNA sequencing. Further, the isolate was capable of inducing black extrinsic tooth stain in an ex vivo model of sound fallen deciduous teeth.

Recently, natural agents have gained an excessive importance as therapeutic source against oral pathogens because of the safety concern about synthetic agents and the abuse of antibiotics (Lotfy et al. 2018, 2019). Furthermore, natural agents can be applied for the prevention of pathology due to their antibacterial activities and can also be used as auxiliary therapy to prevent systemic infections caused by transient bacteraemia during dental procedures, sub-clinical dental infection or daily dental brushing (Potron et al. 2010; Rahamat‐Langendoen et al. 2011; Roda et al. 2008; Wang et al. 2010). Therefore, there is a need to search new natural bioactive compound that should demonstrate broad approach of action targeting effectively both free- and biofilm-mode of *Aggregatibacter actinomycetemcomitans* cells. In the literature, the antibacterial effect of *Hypericum perforatum* (Arpag et al. 2020) essential oil and coconut oil (Baharvand et al. 2021) on *Aggregatibacter actinomycetemcomitans* was reported. The antibacterial activity of cinnamon essential oil against *Staphylococcus aureus*, *Escherichia coli* and *Pseudomonas aeruginosa* was also investigated (El Atki et al. 2019). Recently, the effect of the ethanolic extract of cinnamon on biofilm formation of *Porphyromonas gingivalis* and *Aggregatibacter actinomycetemcomitans* was investigated (Panjaitan et al. 2022). The current work focused on the study of cinnamon essential oil and its individual components against planktonic and sessile *Aggregatibacter actinomycetemcomitans*. To our knowledge, no study has reported the in vitro effect of cinnamon essential oil and its individual compounds on *Aggregatibacter actinomycetemcomitans*.

The chemical analysis of cinnamon essential oil using gas chromatograph–mass spectrometer has revealed eight compounds. Out of these compounds, only eugenol, cinnamaldehyde and α-methyl cinnamaldehyde demonstrated anti-*Aggregatibacter actinomycetemcomitans* activities. Eugenol exhibited the lowest minimum inhibitory concentration and the values of the minimum inhibitory concentrations were in the following order: cinnamon essential oil (421.5 mg/ml) > α-methyl cinnamaldehyde (26.37 mg/ml) > cinnamaldehyde (0.209 mg/ml) > eugenol (0.052 mg/ml). In another study, the ethanolic extract of cinnamon with a concentration of 2.5% was effective against biofilm formation of *Aggregatibacter actinomycetemcomitans*. It is likely that the low minimum inhibitory concentration values in this study are due to the difference in chemical composition and physical properties of cinnamon essential oil compared to the ethanolic extract of cinnamon. With respect to the antibacterial activity, eugenol and cinnamaldehyde exert it by permeabilising the cell membrane and interacting with proteins causing leakage of adenosine triphosphate and potassium ions from the cell (Di Pasqua et al. 2007).

Cinnamon essential oil, eugenol and cinnamaldehyde, respectively, exhibited two-, four- and fourfold increase of sessile minimum inhibitory concentration compared to their planktonic minimum inhibitory concentration. This finding clearly showed that cinnamon essential oil, eugenol and cinnamaldehyde are equally effective against both planktonic and sessile modes of *Aggregatibacter actinomycetemcomitans*. Moreover, they have an added value over ceftriaxone to which several-fold increase in biofilm resistance was perceived. These results are in agreement with Jafri et al. (2019) who studied the in vitro efficacy of eugenol on single and mixed biofilms of *Streptococcus mutans* (Jafri et al. 2019). However, the results of cinnamaldehyde contradict the findings of Chung et al. (2018) who found that cinnamaldehyde has no effect on the adhesion and internalisation of *Aggregatibacter actinomycetemcomitans* (ATCC 33384) to THP-1 cells (Chung et al. 2018). This divergence is more likely due to the difference in biofilm susceptibility to cinnamaldehyde between the two examined *Aggregatibacter actinomycetemcomitans* strains which has been previously reported (De Martino et al. 2009). Further, cinnamaldehyde has multiple targets of activity that can influence strains differently (Nazzaro et al. 2013) as it can inhibit the growth of some Gram-negative bacteria without degeneration of the outer membrane or depletion of intracellular adenosine triphosphate (Nazzaro et al. 2013).

The growth kinetics of both planktonic and sessile *Aggregatibacter actinomycetemcomitans* in the presence or absence of cinnamon essential oil, eugenol, cinnamaldehyde and α-methyl cinnamaldehyde was also investigated. A delayed start of the lag phase of planktonic *Aggregatibacter actinomycetemcomitans* by 36 and 32 h was observed at half the minimum inhibitory concentrations of cinnamon essential oil and eugenol, respectively. Both planktonic and sessile *Aggregatibacter actinomycetemcomitans* cells were completely inhibited at the minimum inhibitory concentrations of cinnamon essential oil, eugenol, cinnamaldehyde and α-methyl cinnamaldehyde. The inhibition of *Aggregatibacter actinomycetemcomitans* biofilm formation by cinnamon essential oil, eugenol, cinnamaldehyde and α-methyl cinnamaldehyde at their sub-minimum inhibitory concentrations ranged from 5.3% to 37.4%. These findings are superior to other studies on the effect of coconut oil against *Aggregatibacter actinomycetemcomitans* (Baharvand et al. 2021), *Actinomyces* sp. (Lavine et al. 2018) and *Prevotella* sp. (Gayatri et al. 2017).

Cinnamon essential oil, eugenol and cinnamaldehyde exhibited inhibitory effect against planktonic and sessile *Aggregatibacter actinomycetemcomitans* cells which is indicated by the reduction in the survival rate after 15–20 and 25–30 min, respectively. These data suggest that cinnamon essential oil, eugenol and cinnamaldehyde could be used to prevent biofilm formation by *Aggregatibacter actinomycetemcomitans* after removal of black extrinsic tooth stain or they could be used as ancillary therapy in preventing bacteraemia of *Aggregatibacter actinomycetemcomitans*. Furthermore, cinnamon essential oil, eugenol and cinnamaldehyde are non-toxic and safe for human use at their minimum inhibitory concentration and minimum bactericidal concentration (Singh et al. 2009). Consequently, if they can be incorporated into children’s chewing gums or mouth wash formulations, this will provide a beneficial effect in preventing the adhesion of *Aggregatibacter actinomycetemcomitans* to tooth enamel and accordingly preventing black extrinsic tooth stain. In contrast, α-methyl cinnamaldehyde showed a better anti-planktonic than anti-biofilm activity. It seems that the presence of methyl moiety in the structure of α-methyl cinnamaldehyde appears to decrease its activity when compared to cinnamaldehyde. In this case, we propose that it could be a good choice to prevent dissemination of *Aggregatibacter actinomycetemcomitans* in the oral cavity or it could be used as an auxiliary therapy in anatomical areas with high risk of bacteraemia. Since various bacteria have been reported to be associated with black extrinsic tooth stain formation (França-Pinto et al. 2012; Slots 1974; Soukos et al. 2005), an ex vivo model was employed using a polymicrobial culture of *Aggregatibacter actinomycetemcomitans*, *Prevotella intermedia* and *Porphyromonas gingivalis* to evaluate the preventive effect of cinnamon essential oil and its active components on the formation of black extrinsic tooth stain. The results revealed that the daily addition of cinnamon essential oil, eugenol and cinnamaldehyde at their minimum inhibitory concentrations for 14 days, totally prevented the formation of black extrinsic tooth stain and, respectively, reduced the survival of sessile bacterial cells to 0.57%, 0.75% and 0.87%. However, the daily incorporation of α-methyl cinnamaldehyde at its minimum inhibitory concentration was not capable of preventing black extrinsic tooth stain formation. Furthermore, α-methyl cinnamaldehyde reduced sessile bacterial cells by 17.63% and black extrinsic tooth stain was visually detectable in the ex vivo model after 10 days of exposure to α-methyl cinnamaldehyde.

A few limitations were encountered in this study. Since the oral cavity is a challenging environment for antimicrobials due to the effects of saliva and the gingival crevicular fluid, further studies will be needed to study the in vivo substantivity of cinnamon essential oil, eugenol, cinnamaldehyde and α-methyl cinnamaldehyde. Furthermore, it is not possible for one study to investigate all the bacterial species associated with black extrinsic tooth stain. Accordingly, future researches should challenge the in vitro effect of cinnamon essential oil, eugenol, cinnamaldehyde and α-methyl cinnamaldehyde on other bacteria from the polymicrobial community of black extrinsic tooth stain.

## Conclusion

CEO and its individual compounds showed a marked antibacterial activity against *Aa*. Their effective results against planktonic and sessile *Aa* within reasonable time indicated that they can be used to prevent BETS. CEO, eugenol, cinnamaldehyde and α-methyl cinnamaldehyde were very effective against planktonic *Aa*. Moreover, CEO and its bioactive components demonstrated a remarkable anti-biofilm activity against *Aa*. Whereas, α-methyl cinnamaldehyde was nearly ineffective against sessile *Aa*.

## Data Availability

The datasets generated and/or analysed during this study are available in the GenBank repository, accession number MK270928.
